# Healthcare Access and Utilization among Korean Americans: The Mediating Role of English Use and Proficiency

**DOI:** 10.5296/ijssr.v4i1.8678

**Published:** 2016-03

**Authors:** Jiang Li, Annette E. Maxwell, Beth A. Glenn, Alison K. Herrmann, L Cindy Chang, Catherine M. Crespi, Roshan Bastani

**Affiliations:** Department of Health Policy and Management, Fielding School of Public Health, Jonsson Comprehensive Cancer Center, and UCLA Kaiser Permanente Center for Health Equity, University of California Los Angeles, 650 Charles E. Young Drive South, Room A2-125 CHS, Los Angeles, CA 90095, USA, Tel: 1-310-206-8954 jiang.li@ucla.edu; Department of Health Policy and Management, Fielding School of Public Health, Jonsson Comprehensive Cancer Center, and UCLA Kaiser Permanente Center for Health Equity, University of California Los Angeles, 650 Charles E. Young Drive South, Room A2-125 CHS, Los Angeles, CA 90095, USA, Tel: 1-310-794-9282 amaxwell@ucla.edu; Department of Health Policy and Management, Fielding School of Public Health, Jonsson Comprehensive Cancer Center, and UCLA Kaiser Permanente Center for Health Equity, University of California Los Angeles, 650 Charles E. Young Drive South, Room A2-125 CHS, Los Angeles, CA 90095, USA, Tel: 1-310-206-9715 bglenn@ucla.edu; Department of Health Policy and Management, Fielding School of Public Health, Jonsson Comprehensive Cancer Center, and UCLA Kaiser Permanente Center for Health Equity, University of California Los Angeles, 650 Charles E. Young Drive South, Room A2-125 CHS, Los Angeles, CA 90095, USA, Tel: 1-310-206-8483 aherrmann@ucla.edu; Jonsson Comprehensive Cancer Center and UCLA Kaiser Permanente Center for Health Equity, University of California Los Angeles, 650 Charles E. Young Drive South, Room A2-125 CHS, Los Angeles, CA 90095, USA, Tel: 1-310-204-9038 clichang@ucla.edu; Department of Biostatistics, Fielding School of Public Health, Jonsson Comprehensive Cancer Center, and UCLA Kaiser Permanente Center for Health Equity, University of California Los Angeles, 650 Charles E. Young Drive South, Room A2-125 CHS, Los Angeles, CA 90095, USA, Tel: 1-310-206-9364 ccrespi@g.ucla.edu; Department of Health Policy and Management, Fielding School of Public Health, Jonsson Comprehensive Cancer Center, and UCLA Kaiser Permanente Center for Health Equity, University of California Los Angeles, 650 Charles E. Young Drive South, Room A2-125 CHS, Los Angeles, CA 90095, USA, Tel: 1-310-206-9266 bastani@ucla.edu

**Keywords:** Acculturation, Mediation, Health Insurance, Asian Americans, Health Disparities

## Abstract

The literature suggests that Korean Americans underutilize health services. Cultural factors and language barriers appear to influence this pattern of low utilization but studies on the relationships among length of stay in the US, English use and proficiency, and utilization of health services among Korean Americans have yielded inconsistent results. This study examines whether English language use and proficiency plays a mediating role in the relationships between length of stay in the US and health insurance coverage, access to and use of care. Structural equation modeling was used for mediation analysis with multiple dependent variables among Korean Americans (N = 555) using baseline data from a large trial designed to increase Hepatitis B testing. The results show 36% of the total effect of proportion of lifetime in the US on having health insurance was significantly mediated by English use and proficiency (indirect effect = 0.166, SE = 0.07, p<.05; direct effect = 0.296, SE = 0.13, p<.05). Proportion of lifetime in the US was not associated with usual source of care and health service utilization. Instead, health care utilization was primarily driven by having health insurance and a usual source of care, further underscoring the importance of these factors. A focus on increasing English use and proficiency and insurance coverage among older, female, less educated Korean Americans has the potential to mitigate health disparities associated with reduced access to health services in this population.

## 1. Introduction

As the fastest-growing group in the United States, the Asian American population is projected to nearly double by 2060, accounting for 11.7% of the total population (Colby & Ortman, 2015). Research suggests that Asian Americans tend to underutilize health services (Derose, Escarce, & Lurie, 2007; Flaskerud & Kim, 1999; S. Lee, Choi, & Jung, 2014; Pourat, Kagawa-Singer, Breen, & Sripipatana, 2010), because of cultural and language barriers, limited knowledge of the United States (US) health care system and social services, lack of access, and lack of trust in the system (Flaskerud & Kim, 1999; Kang et al., 2010). Some of these challenges are due to the process of acculturation and life changes experienced by Asian American immigrants. (Derose et al., 2007; Flaskerud & Kim, 1999; S. Lee et al., 2014; Pourat et al., 2010)

Compared to other Asian ethnic groups, Korean Americans stand out as having one of the lowest rates of health care utilization (Maxwell & Crespi, 2009; Maxwell, Crespi, Antonio, & Lu, 2010; Pourat et al., 2010; Ryu, Young, & Park, 2001). Previous studies have examined factors associated with having a usual source of care (S. Lee et al., 2014), mental health service utilization (Park, Cho, Park, Bernstein, & Shin, 2013), cancer screening (H. Y. Lee, Ju, Vang, & Lundquist, 2010), ambulatory care utilization (Shin, Song, Kim, & Probst, 2005; Sohn & Harada, 2004) and use of alternative medicine (J. Kim & Chan, 2004) among Korean Americans. These studies suggest that length of stay in the United States, having health insurance and having a usual source of care are among the most important factors associated with receiving health services among Korean Americans. However, most of these previous studies have focused only on use of a particular health service, and not on use of health services more generally (J. Kim & Chan, 2004; H. Y. Lee et al., 2010; S. Lee et al., 2014; Park et al., 2013; Shin et al., 2005; Sohn & Harada, 2004).

Length of stay in the US is often considered a marker for acculturation, which is the process of adopting the attitudes, values, customs, beliefs, and behaviors of another culture (Abraido-Lanza, Armbrister, Florez, & Aguirre, 2006; Lopez-Class, Castro, & Ramirez, 2011). As a learning and communication process, acculturation is perceived as easier during early childhood and increases with increasing length of time spent in the US. Compared to immigrants who arrive in the US at a young age, older immigrants are more likely to experience challenges in acculturation due to limited English skills, little or no US work experience, and weak ties to social networks (Scommegna, 2013), which in turn could result in reduced health care access and utilization. Few studies have examined the relationships between length of stay in the US, English use and proficiency and utilization of general (not specialized) health services among Korean Americans, and they have yielded inconsistent results (Frisbie, Cho, & Hummer, 2001; J. Kim & Chan, 2004; Shin et al., 2005). While some studies have found statistically significant positive relationships between length of stay in the US, English use and proficiency and health service use (Frisbie et al., 2001; J. Kim & Chan, 2004), others have found that these variables were not critical in explaining utilization of health services among Korean Americans (Shin et al., 2005). Only one of these studies has examined the role of length of stay in a life course framework (i.e., the ratio of years of stay in the US to age, or proportion of lifetime in the US) (Shin et al., 2005).

Our study uses the Andersen Behavioral Model of Health Services Use (R. Andersen & Davidson, 2007) to examine the relationships among individual predisposing (age, gender, education, length of stay in the US), enabling (English use and proficiency, health insurance, usual source of care), and need factors (self-reported health status) as well as their effects on health services utilization in a large sample of Korean Americans in Los Angeles County which is home to the largest number of ethnic Koreans outside of Korea. Andersen R. M. argued that the behavioral model suggested explanatory processes or causal ordering where the predisposing factors might be exogenous, enabling resources were necessary but not sufficient conditions for use (R. M. Andersen, 1995), implying mediation relationships among predisposing, enabling factors and use. Only a few studies have explicitly tested these relationships (Stein, Andersen, Robertson, & Gelberg, 2012). To date, no study has empirically tested whether language barriers play a role in the relationship between length of stay in the US and health care access and utilization. Our study aimed to fill this research gap by testing the following hypotheses: a) Korean Americans with increasing length of stay in the US are more likely to have health insurance, a usual source of care and have visited a doctor (or traditional practitioner) in the past year; b) English use and proficiency mediates the relationships between length of stay in the US and health care access and utilization.

## 2. Methods

### 2.1 UCLA Korean Healthy Life Project

The UCLA Korean Healthy Life Project was a randomized controlled trial designed to evaluate an intervention to increase Hepatitis B (HBV) testing among Koreans Americans (Bastani et al., 2015). Eligible participants were 18–64 years of age, of Korean ethnicity, Korean or English speaking, who had no history of HBV testing. The sample for this study was limited to 555 participants recruited from 28 Korean churches in Los Angeles between 2007 and 2010 who provided baseline data on health care access and utilization. The church and participant recruitment are described elsewhere (Bastani et al., 2015). The UCLA Korean Healthy Life Project was approved by the Institutional Review Board at the University of California, Los Angeles.

### 2.2 Measures

In-person interviews were conducted at churches in Korean language assessing: demographic characteristics (age, marital status, education, income), age at immigration, health care access (health insurance, usual source of care), recent health care utilization, and self-reported general health. To assess the recent health care utilization, participants gave Yes/No responses on two questions-“During the past 12 months, did you see a doctor or health professional about your own health?” and “During the past 12 months, did you see a traditional practitioner about your own health?” Both having visited a doctor and a traditional practitioner, which were not mutually exclusive, were included in the study as measures of health care utilization. Proportion of lifetime in the US was calculated by years of stay in the US divided by age and entered into all statistical models as a continuous variable. The mediator English use and proficiency was assessed using four Likert-type items: one item for English proficiency and three items adapted from the short Marin Acculturation Scale to assess English use when communicating with friends, reading newspapers/magazines, and watching TV (Marin, Sabogal, Marin, Otero-Sabogal, & Perez-Stable, 1987). The Internal Consistency reliability Cronbach Coefficient Alpha of the 4-item scale is 0.85.

### 2.3 Conceptual Framework

**Figure 1:**
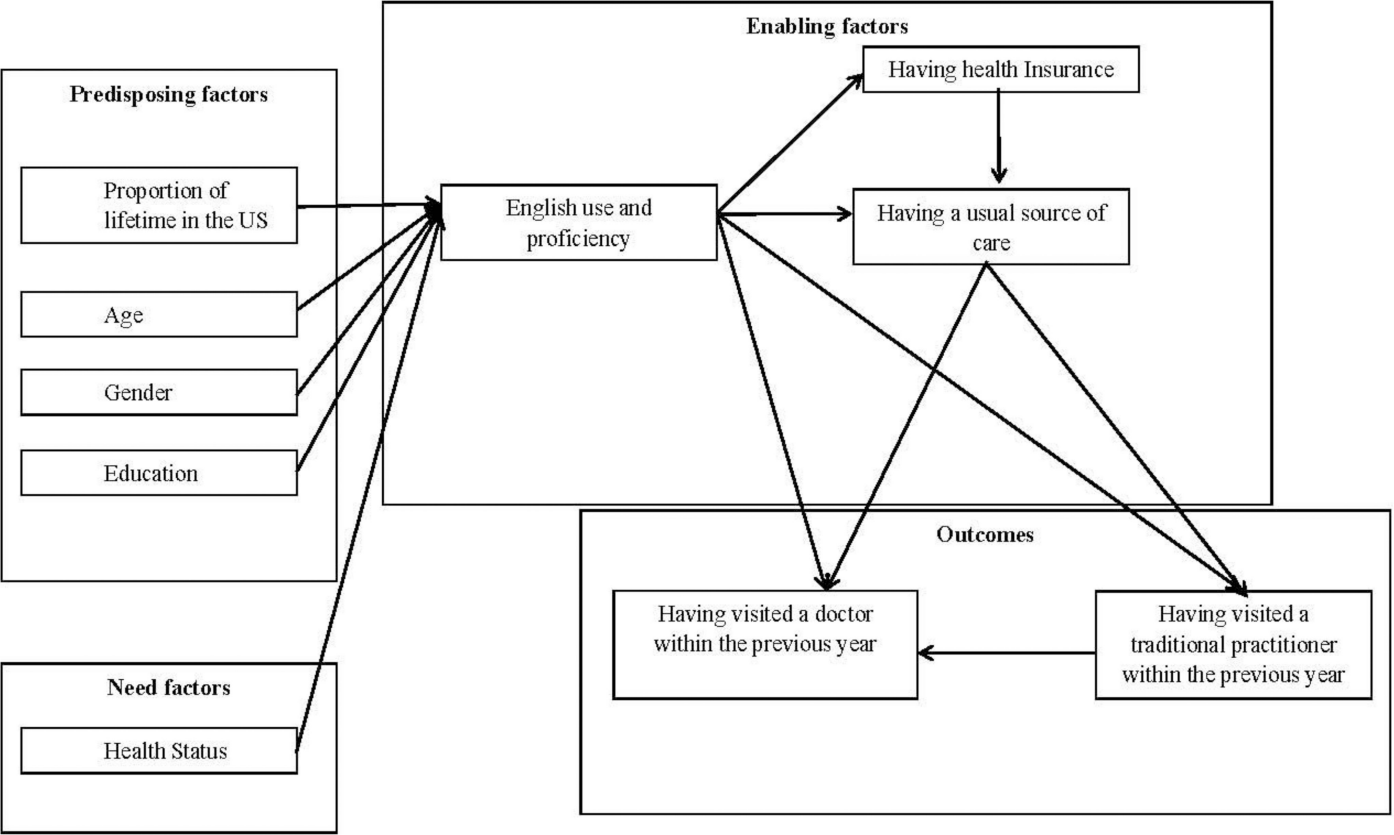
Conceptual model of acculturation and health services utilization among Korean Americans *Note:* In order to simplify demonstration of the model, the hypothesized paths from predisposing factors to enabling factors other than English use and proficiency, need factors and outcomes are not shown, similarly, the paths from need factors to outcomes are not shown.

Suggested by the Andersen Behavioral Model of Health Services Use (Figure 1), the present study examined the role of predisposing factors (age, gender, education, proportion of lifetime in the US), potential health needs (self-reported health status), and enabling factors (English use and proficiency, having health insurance, having a usual source of care) in health services utilization (having visited a doctor or traditional practitioner within the previous year) with a strong emphasis on measures of acculturation in a life course perspective (Abraido-Lanza et al., 2006; Lopez-Class et al., 2011) (proportion of lifetime in the US, English use and proficiency). The requirements for establishing mediation include the assumption of causal order (James & Brett, 1984). In a cross-sectional data set, we test if English use and proficiency is a mediator between proportion of lifetime in the US and health care access and utilization, which is appropriate given the nature of the independent variable, mediator and dependent variables. Specifically, proportion of lifetime in the US is calculated based on age and age at immigration, both of which are not influenced by English use and proficiency at the time of assessment. In addition, English use and proficiency are unlikely to be influenced by health care access and utilization.

### 2.4 Data Analysis

Analyses of sample characteristics along with correlation among variables were conducted using SAS 9.3. Structural equation modeling (Mplus 6.12, Muthen & Muthen, 2011) was used to test the hypotheses simultaneously using Maximum Likelihood estimation with Monte Carlo integration. The mediator (English use and proficiency) was operationalized as a latent variable measured by multiple indicators (4 items). The measurement error in the mediator causes an underestimated indirect effect and an overestimated direct effect (Hoyle & Kenny, 1999; le Cessie, Debeij, Rosendaal, Cannegieter, & Vandenbroucke, 2012; Vander Weele, Valeri, & Ogburn, 2012). The latent variable model gives very small biases compared to the sums of items as the mediator (Muthen & Asparouhov, 2015). Therefore, a series of models were specified and tested to determine if English use and proficiency was a significant mediator of the relationships between proportion of lifetime in the US and health care access and utilization while controlling for demographic variables. Prior to the analyses of the structural model, a measurement model was examined to identify the latent variable –English use and proficiency (Bowen, 2012) and found all factor loadings were above 0.88, indicating that the four indicators (items) were reliable measures of their latent construct- English use and proficiency (Bowen, 2012).

The first structural model (Model 1) evaluated the effects of proportion of lifetime in the US on health care access (insurance and usual source of care) and utilization outcomes controlling for demographic variables. A second model (Model 2) was tested to simultaneously evaluate the effects of proportion of lifetime in the US on health insurance and utilization outcomes and their effects mediated by English use and proficiency (MacKinnon, 1994) adding paths between the latent variable and proportion of lifetime in the US (linear regression estimate) and the latent variable and demographic variables and outcomes (logistic regression estimates). We relied on the Akaike Information Criterion (AIC) (Akaike, 1987), the Bayesian Information Criterion (BIC) (Schwarz, 1978), and the sample size-adjusted Bayesian Information Criterion (ssaBIC) (Sclove, 1987) with lower values indicating better model fit. These fit statistics and the Satorra-Bentler scaled Chi-square different test were used to determine the optimum model (Satorra, 2000). Based on the method by Breen et al. (Breen, Karlson, & Holm, 2013), the total effect in logits was decomposed into a direct part and an indirect part. Indirect effects and their statistical significance were also derived from Mplus 6.0 (Muthen & Muthen, 2010). The proportion mediated is a ratio of the significant indirect effect to the total effect (MacKinnon, 1994). Both models also take into account non-independence of observations across churches (clusters).

## 3. Results

### 3.1 Sample Characteristics

Sample characteristics are presented in Table 1. Compared to the overall LA County Korean population based on 2007–2009 American Community Survey 3-year estimates, our sample had a higher proportion of females (65% vs. 53% in LA County), college graduates (54.4% vs. 37.9% in LA County), and immigrants (97.3% vs.76.1% in LA County). Approximately half (46.7%) of the participants had an income of less than $50 000 (compared with the median household income of the overall LA county Korean population which is $50 075). Fifty-nine percent of the participants lacked insurance, 50% had a usual source of care, 53% reported that they had visited a doctor within the previous year and 34% had visited a traditional practitioner within the previous year.

### 3.2 Structural Equation Models

**Figure 2:**
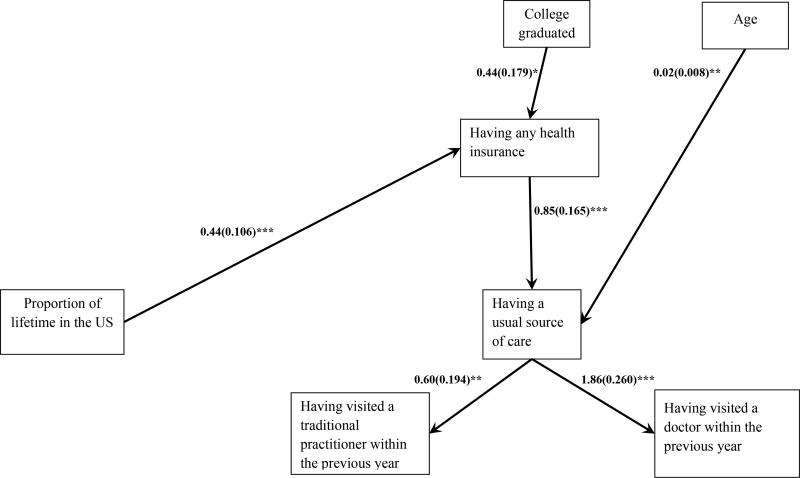
Model 1 - Effects of proportion of lifetime in the US on health care access and utilization. Note: Model is adjusted for demographic variables. * p<.05, ** p<.01, *** p<.001. The numbers beside the lines indicate path coefficient values and standard errors (Odds Ratios and nonsignificant paths are not shown in the model).

Model 1 (AIC = 7506.691, BIC = 7643.962, ssaBIC = 7542.383) estimated the direct effects of the proportion of lifetime in the US on health care access and utilization controlling for demographics variables (Figure 2). In Model 1, for a 25% increase in proportion of lifetime in the US, there was 55% increase in the odds of having health insurance (OR = 1.55, 95%CI = 1.26 – 1.91). Proportion of lifetime in the US also had a positive impact on usual source of care and doctor visits and a negative impact on having visited a traditional practitioner, but the effects were not statistically significant.

**Figure 3:**
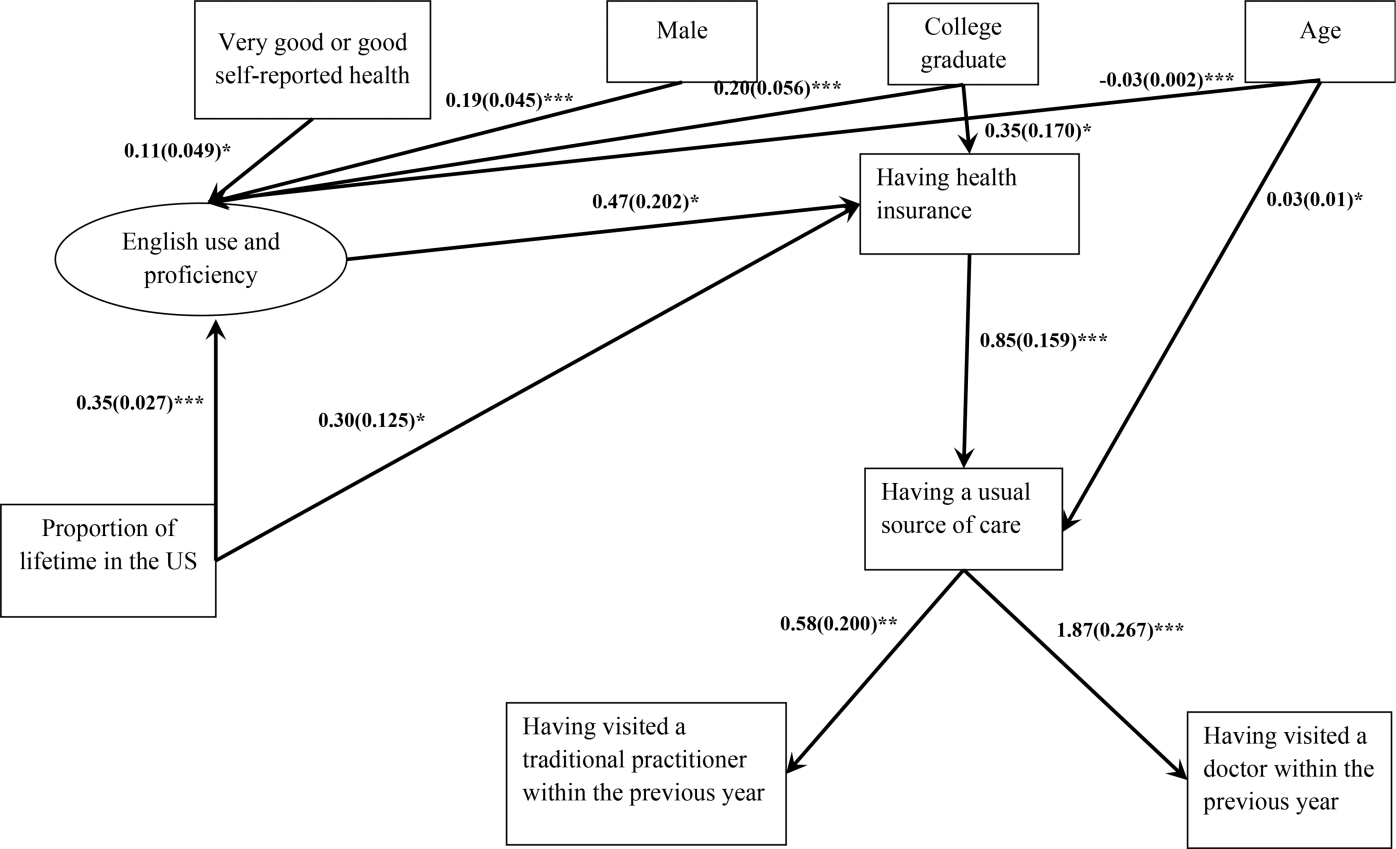
Model 2 - Mediated effects of proportion of lifetime in the US on health care access and utilization *Note:* Model is adjusted for demographic variables. * p<.05, ** p<.01, *** p<.001. The numbers beside the lines indicate path coefficient values and standard errors (Odds Ratios and nonsignificant paths are not shown in the model).

The addition of the English use and proficiency mediator variable controlling for demographic variables (Model 2: AIC = 7168.321, BIC = 7344.199, ssaBIC = 7214.051, ΔLoglikelihood = 345.55, Δdf = 9, p<.001) made significant improvements to the overall model fit. As shown in Model 2 (Figure 3), being younger (β = −0.03, SE = 0.002, p<.001), male (β = 0.19, SE = 0.05, p<.001), having a college degree (β = 0.2, SE = 0.06, p<.001) and reporting better health status (β = 0.11, SE = 0.05, p<.05) was related to higher levels of English use and proficiency compared to being older, female, less educated, and reporting lower health status. Proportion of lifetime in the US was significantly positively associated with English use and proficiency (β = 0.35, SE = 0.03, p<.001) and having health insurance (OR = 1.34, 95%CI = 1.06 – 1.72). The addition of the English use and proficiency mediator to the model led to an overall reduction of the direct effect between proportion of lifetime in the US and having any health insurance. For a one-unit increase in English use and proficiency, there was 60% increase in the odds of having health insurance (OR = 1.6, 95%CI = 1.07 – 2.37). English use and proficiency significantly mediated the relationship between proportion of lifetime in the US and having health insurance (Indirect effect = 0.166, SE = 0.07, p<.05; Direct effect = 0.296, SE = 0.13, p<.05; Total effect = 0.462, SE = 0.12, p<.001). The magnitude of the indirect effect through English use and proficiency is around 56% of the magnitude of the direct effect of proportion of lifetime in the US on health insurance (0.166/0.296) and accounts for 36% of the total effect of proportion of lifetime in the US on health insurance in the logit model (0.166/0.462).

For Korean Americans with health insurance, the odds of having a usual source of care were 2.33 times (95%CI = 1.71 – 3.19) the odds for those without health insurance. Older Korean Americans were more likely to have a usual source of care (OR = 1.03, 95%CI = 1.00 – 1.10). For Korean Americans with a usual source of care, the odds of having visited a doctor and a traditional practitioner within the previous year were 6.47 times (95%CI = 3.83 – 10.92) and 1.79 times (95%CI = 1.21 – 2.64) the odds for those without a usual source of care, respectively (ORs and 95% CI not shown in the figures).

## 4. Discussion

Public health research on acculturation has traditionally relied upon linear assimilation models, in which important constructs underlying the developmental processes such as age at immigration or proportion of lifetime in the US have been understudied (Abraido-Lanza et al., 2006; Lopez-Class et al., 2011). This study provides a more nuanced perspective by examining the mediating role of English use and proficiency in the relationship between proportion of lifetime in the US and health care access and use in one Asian American group with very low rates of health services utilization. Our results indicate that the proposed model of acculturation and health services utilization explains the cross-sectional data from a sample of Korean Americans reasonably well.

Because the history of Korean Americans in the US is relatively shorter than other American ethnic groups (Y. Y. Kim, 1978), most Korean Americans are immigrants or the children of immigrants for whom acculturation marks many aspects of their lives including access and health services utilization. A systematic review of colorectal cancer screening studies among Korean Americans revealed that Korean Americans with longer length of stay and more acculturation generally had higher screening rates (Oh & Jacobsen, 2014). Our findings support previous research by highlighting that a higher proportion of lifetime in the US was significantly associated with having health insurance as well as higher level of English use and proficiency. This finding is also consistent with existing evidence that links length of stay in the US with health insurance coverage (Nam, 2008; Vargas Bustamante et al., 2014).

Furthermore, we found that English use and proficiency was influenced by age, sex, education, and self-reported health. After adjusting for sociodemographic factors, the mediating role of English use and proficiency remained significant. Our study adds the new observation that about one third of the total effect of proportion of lifetime in the US on having health insurance was significantly mediated by English use and proficiency. However, the data do not support the hypotheses that a higher proportion of lifetime in the US and English use and proficiency were associated with having a usual source of care or having visited a doctor (or traditional practitioner) in the previous year. Instead, health care utilization was primarily driven by having health insurance and a usual source of care, further underscoring the importance of having health insurance and a usual source of care.

Nearly sixty percent of the participants lacked insurance, which is comparable to the percentage (64.3%) of uninsured Korean Americans found in the 2009 population-based California Health Interview Survey (S. Lee et al., 2014). Based on the same report, Korean Americans were less likely to be insured than their Chinese and Vietnamese counterparts (S. Lee et al., 2014). Consistent with previous studies (Akresh, 2009; Chang, Chan, & Han, 2015), immigration-related factors including length of stay in the US and English use and proficiency are key drivers of disparities in health insurance coverage and should be included in access-to-care models with Asian Americans.

Although we were able to quantify the mediating role of English use and proficiency in the relationship between proportion of lifetime in the US and health care access and utilization, there are several limitations. First, cross-sectional data limit our ability to draw conclusions about the causality of the observed relationships. Second, this study is unable to account for the social and political context in which the immigrant lives. For example, we did not measure the immigration status of participants (e.g., undocumented immigrants, naturalized citizen) or availability of local health services in Korean language. Finally, all participants were members of Korean churches in the Los Angeles region, which limits generalizability of findings to non-church going Korean Americans or Korean Americans residing in other parts of the country.

## 5. Conclusion

This paper draws attention to older, less educated Korean Americans, new immigrants, and Korean American women who may need more help to overcome language barriers, to navigate the American healthcare system and to apply for health insurance. With increasing length of stay in the US, Korean Americans gain English proficiency and familiarity with the health care system, resulting in increased access to health insurance. Still, two-thirds of the total effect of proportion of lifetime in the US on health insurance observed in our study was not attributable to English use and proficiency. Other factors such as immigration status, affordability, and job eligibility may be additional important barriers to obtaining health care coverage (Vargas Bustamante et al., 2014).

Our study was conducted before the passage of the Affordable Care Act (ACA) which expands access to health insurance coverage, including for immigrants who have been in the US for five or more years. Therefore, future studies may be needed to understand whether the relationships and findings uncovered in this study hold in the rapidly changing health insurance environment.

## Figures and Tables

**Table 1:** Characteristics of 555 Korean Americans recruited from 28 Korean churches in Los Angeles from 2007–2010

	N	%
Female	362	65.2
Immigration category		
Foreign-born and living in the US for below 1/4 of life	213	38.5
Foreign-born and living in the US for 1/4–1/2 of life	213	38.5
Foreign-born and living in the US for 1/2–3/4 of life	106	19.1
Foreign-born and living in the US for over 3/4 of life	8	1.4
US born	14	2.5
Married	404	73.1
Education		
Did not complete high school	18	3.3
High school graduate	148	26.8
Some college	85	15.4
College graduate	302	54.6
Total household income		
< $50,000	259	46.7
>$50,000	196	35.3
DK/RF	100	18.0
Self-reported health status		
Fair/Poor/Very Poor	278	50.6
Good/Very Good	271	49.4
Have any health insurance	228	41.1
Have a usual source of care	273	50.2
Have visited a doctor in the previous year	295	53.2
Have visited a traditional practitioner in the previous year	189	34.1
	Mean (Std)	Range
Age	45.0 (12.4)	18–64
Body Mass Index	22.9 (2.9)	15–34
Proportion of lifetime in the US (years of stay in the US/age)	34.9 (22.1)	0–100
English use and proficiency Scale(4-item 5 point Likert Scale)	2.4 (0.8)	1–5
English use with most of friends	2.0(0.9)	1–5
English use when reading newspapers or magazines	2.1(1.0)	1–5
English use when watching TV	2.5(1.0)	1–5
English proficiency	2.9(0.9)	1–5
